# Recurrent COVID-19 Infection in a Refractory/Classical Hodgkin's Lymphoma Patient Undergoing Autologous Stem Cell Transplantation: A Case Report

**DOI:** 10.7759/cureus.46950

**Published:** 2023-10-13

**Authors:** Munerah Hamed, Doaa Alamoudi

**Affiliations:** 1 Department of Pathology, Faculty of Medicine, Umm Al-Qura University, Makkah, SAU; 2 Department of Pathology and Laboratory Medicine, Division of Molecular Medicine, King Abdulaziz Medical City, National Guard Health Affairs, Jeddah, SAU

**Keywords:** chemoimmunotherapy, asct, reinfection, relapsed/refractory hodgkin’s lymphoma, covid-19

## Abstract

Patients with challenging hematological malignancies like classic Hodgkin lymphoma (cHL) can be further complicated when affected by a concurrent coronavirus disease-2019 (COVID-19) infection and often face unique and complex management and outcomes. In this case report, we describe a refractory or relapsed classic Hodgkin lymphoma patient with a recurrent infection of COVID-19 three times preceding chemotherapy. A 52-year-old female presented to our hospital with a second incidence of COVID-19 and a complaint of fever, anorexia, night sweats, and abdominal lymphadenopathy, for which she was diagnosed with mixed cellularity classic Hodgkin lymphoma. Three weeks later, in consideration of her manifestation of lung disease, which was due to her past medical history of airway hypersensitivity and abnormal pulmonary function test along with testing positive for COVID-19, she was started with the first-line chemotherapy of the brentuximab vedotin, doxorubicin, vinblastine, and dacarbazine chemotherapy regimen, commonly referred to as Bv-AVD, without bleomycin. After six cycles of chemotherapy, at the end of treatment, positron emission tomography/computed tomography (PET/CT) revealed the progression of nodes in the abdomen and the development of new lymphadenopathy in the chest and right supraclavicular region. Hence, it was considered refractory Hodgkin’s lymphoma, and the patient was referred for salvage therapy. She was started on salvage chemotherapy with brentuximab/bendamustine (BvB). Follow-up evaluations after two cycles of BvB continued to show newer lesions in the right sub-diaphragmatic area, internal mammary, and supraclavicular lymph nodes. Therefore, the patient was switched to pembrolizumab immunotherapy, a PD-1 inhibitor. After four cycles of pembrolizumab monotherapy, PET/CT showed significant improvement with a complete molecular response (CMR). Then, she was admitted for high-dose therapy/autologous stem cell transplantation (HDT/ASCT) after collecting stem cells. PET/CT: three months post-ASCT, she continued to be in a CMR with a Deauville score of 1. The patient was continued on pembrolizumab maintenance for six months afterward. Currently, the patient is healthy and doing well. COVID-19 patients with hematological malignancies may experience compromised viral elimination and a prolonged period of viral infection, which may also worsen the symptoms and outcomes and entitle them to comprehensive and extended care.

## Introduction

The coronavirus disease of 2019-the COVID-19 pandemic-has been a challenge for all parts of society across the world. Having been initially described as severe acute respiratory syndrome coronavirus 2 (SARS-CoV-2), the COVID-19 infection has a global resonance and represents a significant threat to several patient populations and health systems. Due to the treatment or the disease itself, cancer patients with immunosuppressive status may have an unfavorable outcome. Moreover, cancer patients are more exposed to serious complications from some infections [1], such as COVID-19, due to their comorbidities and cytotoxic treatments [2]. However, the association between advanced cancer, chemotherapy, and COVID-19 severity is still unclear [3,4].

The majority of patients with hematological malignancies receive anticancer medications that suppress bone marrow function, which affects their immune system, posing a significant risk of both community- and hospital-acquired infections [5]. Patients with lymphoma appear particularly susceptible to COVID-19 infection, partly due to T- or B-cell dysfunction or the harmful effects of the chemotherapy regimens on the immune system [6,7]. Furthermore, the COVID-19 infection caused obstacles for doctors to decide on the treatment regimens of critically ill patients [8]. In addition, most hospitalized individuals with hematological cancers and COVID-19 have an increased mortality rate, which seems to be associated with bacterial co-infection, which aligns with an increased likelihood of reduced granulocyte levels due to their underlying disease or treatment [5]. Subcategories of patients with COVID-19 have been classified as at high risk of morbidity and mortality [9], including patients of older age, male sex (vs. female), and those with comorbidities such as hypertension, chronic lung disease, diabetes, immunodeficiency, and cancer [10]. In particular, cancer patients often follow a more severe and rapid disease course, requiring high-level intensive care and an increased risk of COVID-19-related death. Indeed, after infecting the pneumocytes, COVID-19 triggers intracellular signaling pathways that promote the release of several pro-inflammatory mediators, leading to the recruitment of neutrophils and monocyte macrophages [11].

The existing evidence suggests that the cytokine storm, characterized by an uncontrolled production of inflammation markers that perpetuate an abnormal systemic inflammatory response, is a key factor in developing acute respiratory distress syndrome (ARDS). Chemokines, which are small molecules with strong chemoattractant properties, contribute to the recruitment of immune cells during inflammation [12,13]. Hematological malignancies and/or immunosuppression may lead to prolonged viral shedding in COVID-19 patients, and strict medical precautions and isolation rules should be followed for COVID-19 infection [14]. Chemotherapy must be selected appropriately, especially during COVID-19 infection, due to the immunocompromised status of the patients associated with disease and treatment. Classic Hodgkin lymphoma (cHL) has been shown to be successfully treated with combined chemotherapy. However, for relapsed/refractory Hodgkin’s lymphoma patients, immunotherapy proved to have a good effect as a treatment.

Clinical trials of immune-mediated therapy for Hodgkin’s lymphoma have shown high efficacy, mostly in relapse/refractory settings [15]. Anti-CD30 (Brentuximab) and immune checkpoint inhibitors (Pembrolizumab, PD-1 inhibitor) showed effective responses before and after autologous stem cell transplantation (ASCT), respectively [15,16]. The recent use of brentuximab, which causes lymphopenia and compromises the CD4 T-cell-mediated immune system, resulted in severe disease, including pneumonia and acute respiratory syndrome related to the COVID-19 infection. Prevention and protection measures to minimize the probability of being exposed to COVID-19 are critical, especially in highly immunosuppressive patients. Particular questions remain around the effect of COVID-19 on lymphomas and the interaction between lymphoma-associated and treatment-induced immunosuppression. While it is expected that future research will help fill up these knowledge gaps, the emergence of new COVID-19 variants is likely to necessitate numerous additional studies.

## Case presentation

**Figure 1:**
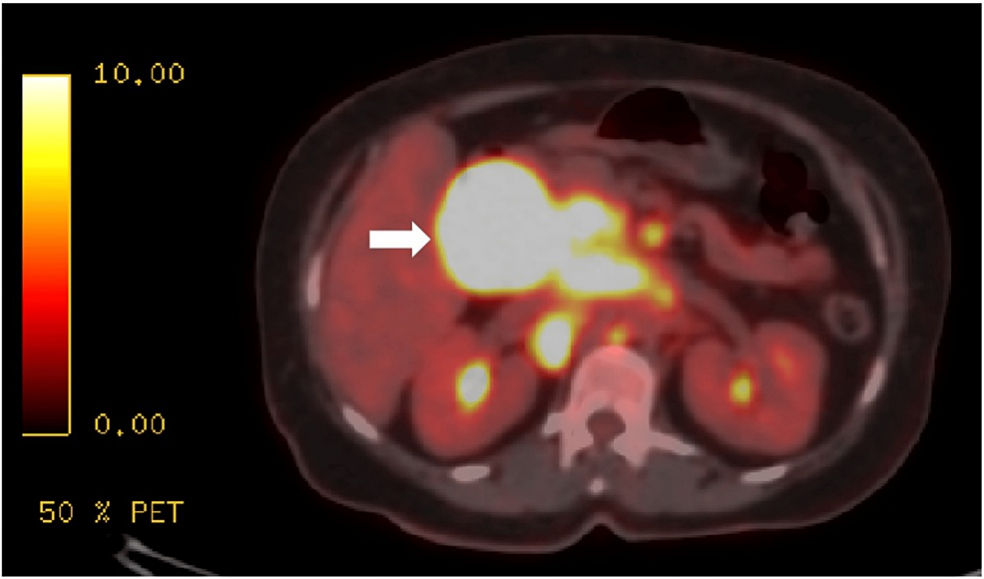
Initial positron emission tomography/CT revealed conglomerated para-aortic and gastrohepatic lymph nodes (the largest porta-hepatis lymph node is visually indicated by a white arrow).

A 52-year-old female was referred to our team at Princess Noorah Oncology Center (PNOC) in October 2020 for fever, anorexia, night sweats, and weight loss for a few months. These symptoms have worsened over the last month before visiting the center. She reported that she had recently tested positive for COVID-19 at another center for the first time in June 2020. Therefore, a repeated test for COVID-19 was performed in our center, where she also tested positive. In addition, she was found to have abdominal lymphadenopathy and worked up for possible lymphoma. She was then admitted for observation and to complete investigations before starting treatment. Due to the concurrent COVID-19 infection, a pulmonary function test was requested, and it was abnormal, revealing lung disease, taking into consideration the past medical history of airway hypersensitivity. A positron emission tomography/computed tomography (PET/CT) scan was done for staging, and it showed conglomerated para-aortic hepatic and gastrohepatic lymph nodes. The largest porta hepatis node measures about 7 X 6.3 X 7.8 cm in transverse (TR), anterior-posterior (AP), and craniocaudal (CC) dimensions, and the maximum standardized uptake value (SUV max) is 18.2. In addition, the largest aortocaval lymph node measures 6.7 X 3.8 cm and has an SUV max of 15.5 cm (Figure 1).

**Figure 2:**
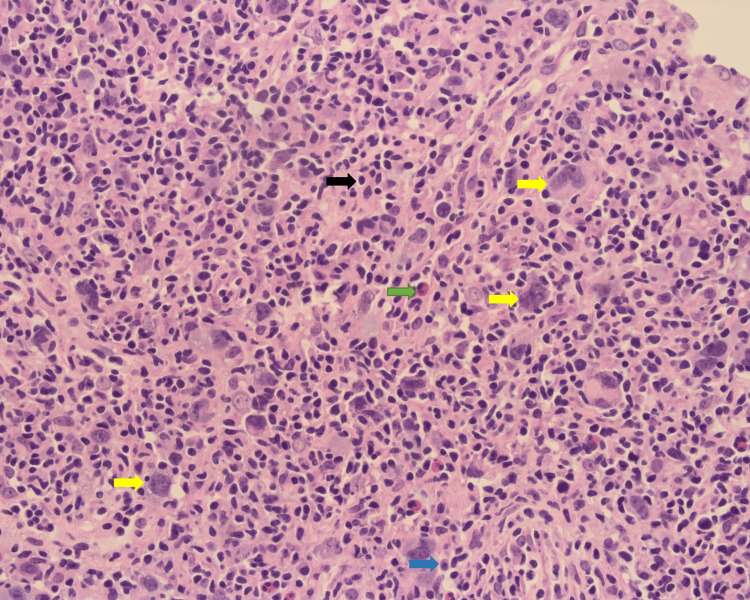
Para-aortic lymph node biopsy revealed many Reed-Sternberg cells (yellow arrows) with mixed chronic inflammatory cells in the background (eosinophils “green arrow”, plasma cells “blue arrow”, and lymphocytes “black arrow”). H&E stained, 40X view (high power).

**Figure 3:**
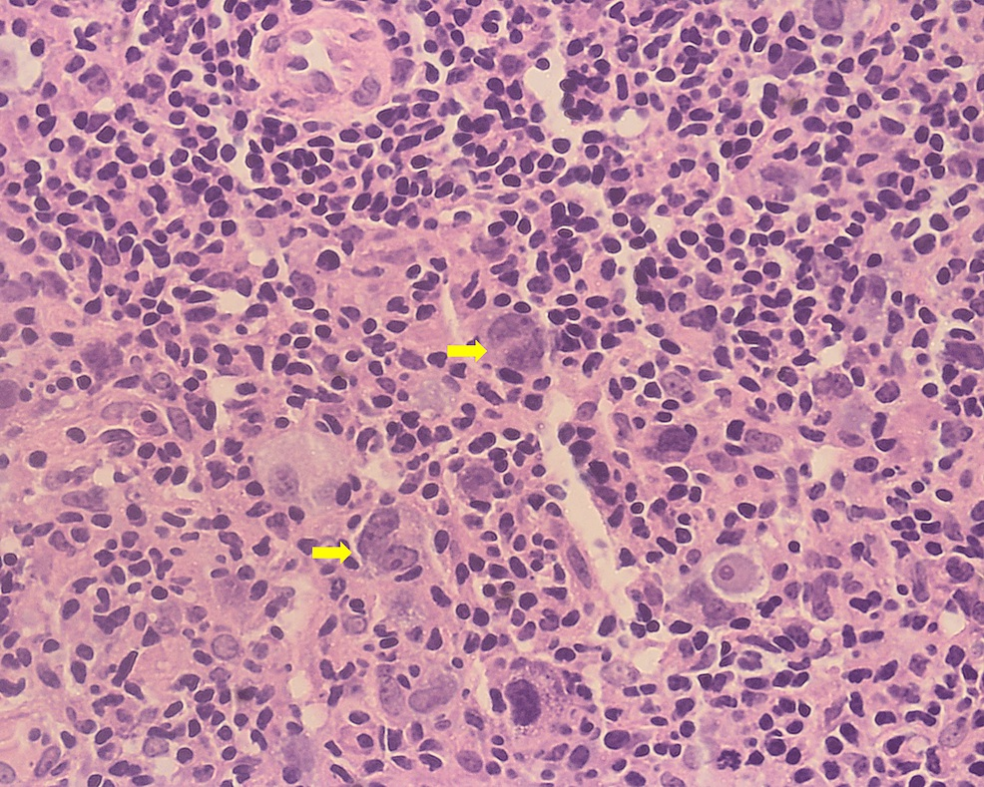
A para-aortic lymph node biopsy showed typical Reed-Sternberg cells (yellow arrows) with mirror image nuclei. H&E stained, 60X view (high power).

**Figure 4:**
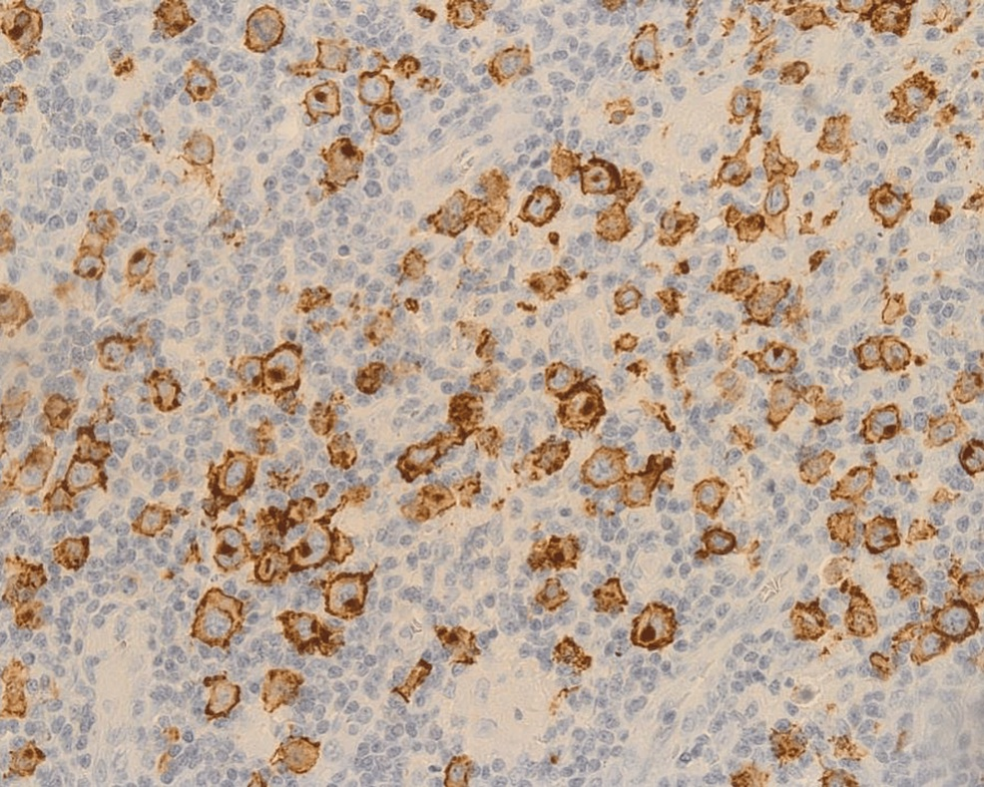
Immunohistochemical staining (IHC) of a para-aortic lymph node biopsy revealed well-demarcated membrane immune labeling for CD30 in the neoplastic cells with and without paranuclear dot-like accentuation of Golgi in the perinuclear zone.

Histopathological examination of para-aortic lymph node biopsy revealed many Reed-Sternberg cells with mixed chronic inflammatory cells in the background (eosinophils, plasma cells and lymphocytes) (Figures 2, 3). Moreover, immunohistochemical staining (IHC) showed strong membrane immune labeling for CD15 and CD30 in the neoplastic cells with and without paranuclear dot-like accentuation of Golgi in the perinuclear zone (Figure 4). Thus, the patient was diagnosed with classic Hodgkin’s lymphoma stage IIIB, mixed cellularity. A bone marrow biopsy revealed no evidence of lymphoma involvement or infiltration. Her past medical history was positive for diabetes, airway hypersensitivity, and allergic rhinitis, and she was on Metformin, the Seretide inhaler, and Montelukast.

Considering the recent positive COVID-19 result occurring within three weeks after the second infection (October 2020), the oncology board regarded this as shedding of infection and thus proceeded with front-line chemotherapy as well as dexamethasone tablets (4 mg for three days). Taking into account her manifestation of lung disease, which was attributed to her past medical history of airway hypersensitivity and abnormal pulmonary function tests, together with testing positive for COVID-19, the decision was made to avoid bleomycin from the standard chemotherapy regimen to avoid lung toxicity. Alternatively, as she had advanced Hodgkin’s lymphoma, she started the chemoimmunotherapy combination as first-line therapy (immune-engaged therapy) of brentuximab vedotin (anti-CD30 antibody-drug conjugate), adriamycin (doxorubicin), vinblastine, and dacarbazine chemotherapy, referred to as Bv-AVD. A blood differential test on the day of the commencement of chemotherapy showed low lymphocyte and neutrophil counts: 0.68 (x109/I) and 0.44 (x109/I), respectively. Therefore, the patient received a subcutaneous injection of filgrastim (300 mcg) for seven days.

Following four cycles of Bv-AVD, laboratory results showed low lymphocyte and neutrophil counts: 0.83 (×109/l) and 1.2 (×109/l), respectively. Oncologists have considered that as febrile neutropenia, which is secondary to her therapy, and she was continued on filgrastim for eight days to stimulate white blood cell (WBC) growth. Interim PET scans after four cycles of Bv-AVD showed an interval decrease in lymph node sizes, but intense metabolic activity was still apparent. Therefore, it was considered a partial response and continued with two more cycles of Bv-AVD.

**Figure 5:**
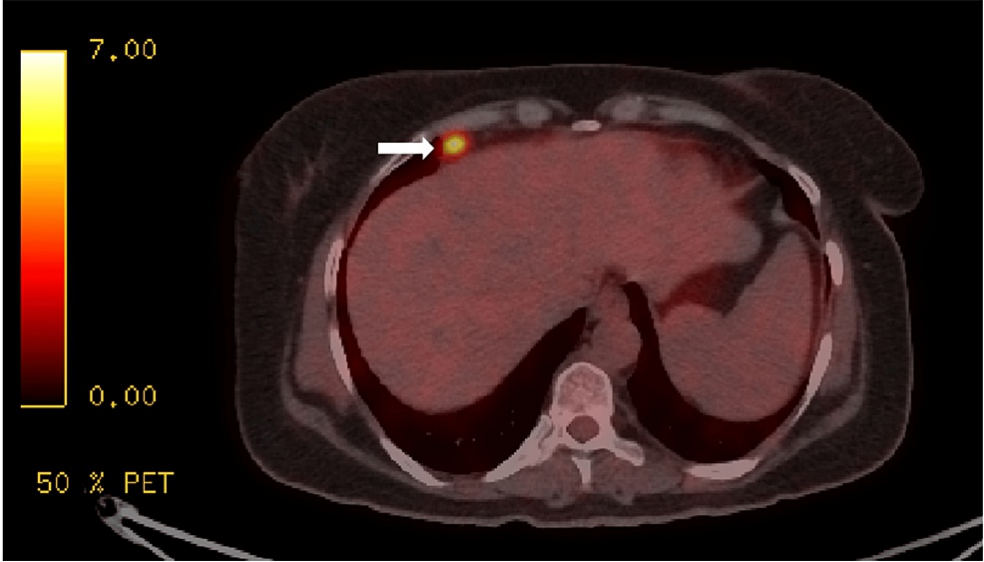
Positron emission tomography/CT revealed new lymphadenopathy in the chest (right sub-diaphragmatic new lymph node visually indicated by a white arrow).

**Figure 6:**
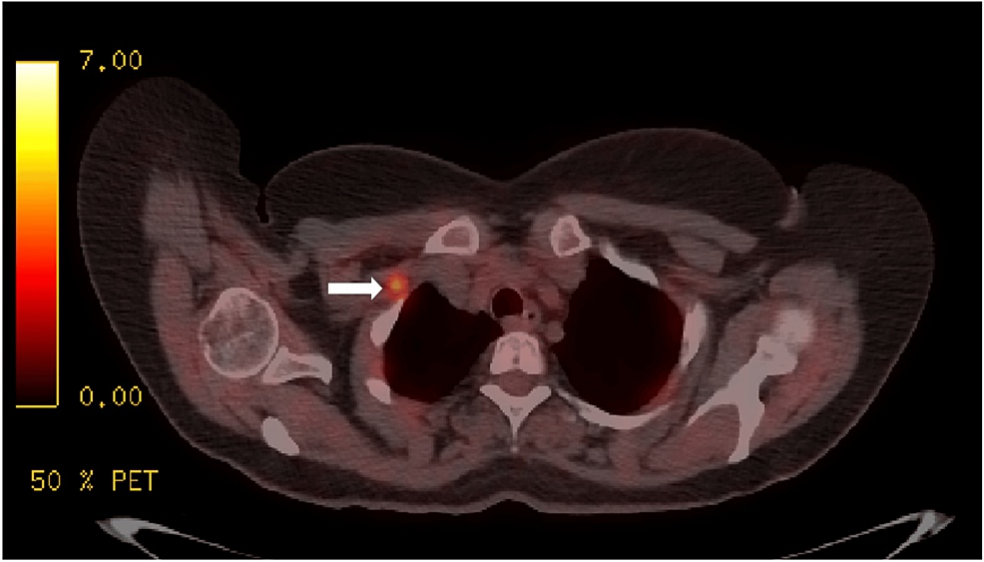
Positron emission tomography/CT showed new lymphadenopathy in the right supraclavicular region, as visually indicated by a white arrow.

At the end of treatment, evaluation showed a new avid right supraclavicular node on the I-157.1 region, which displays an SUV max of 4.5. Moreover, multiple new mediastinal and bilateral hilar nodes showed increased fluorodeoxyglucose (FDG) uptake. An anterior-posterior node was also discovered and measured 1.8 X 0.9 cm with an SUV max 8.7. Some small mild left axillary nodes were also noted. There has been interval significant progression in abdominal nodes seen periportally and in the para-aortic regions. A lymph node in the I-412.2 region measures 4.0 X 2.3 cm with an SUV max 16.4. Previously, the largest node in this region measured 3.8 X 2 cm with an SUV max of 8.3 cm. Hence, Deauville 5 progressive disease with the progression of nodes in the abdomen, new lymphadenopathy in the chest (Figure 5), and the right supraclavicular region (Figure 6). As a result, it was considered primary refractory Hodgkin’s lymphoma; hence, she was started on salvage chemotherapy with brentuximab vedotin/bendamustine (BvB). After two cycles of BvB, follow-up evaluation revealed newer lesions in the right sub-diaphragmatic area, internal mammary, and supraclavicular lymph nodes. Hence, the patient was switched to pembrolizumab immunotherapy. After four cycles of pembrolizumab monotherapy, PET/CT showed significant improvement with a complete molecular response (CMR).

Shortly after, the patient was admitted electively for high-dose therapy/autologous stem cell transplantation (HDT/ASCT) after harvesting stem cells (stem cells were collected 15 days before stem cell infusion). Noteworthy, the patient tested negative for COVID-19 before starting HDT/ASCT. Consequently, she began with the first high-dose therapy of BEAM combination (BCNU, etoposide, Ara‐C, and melphalan) on day 7, continuing until BEAM day 1 according to the European Society for Blood and Marrow Transplantation (EBMT) guidelines. A blood test result revealed neutropenic precaution on BEAM day 1, but stem cell infusion proceeded on BEAM day 0 through a Hickman line. On BEAM day +5, the patient developed febrile neutropenia and was started on Meropenem, as the blood culture result showed Klebsiella pneumonia. Four days later, she experienced a fever spike of 38°C, prompting a change in antibiotics to Meropenem and Vancomycin.

**Figure 7:**
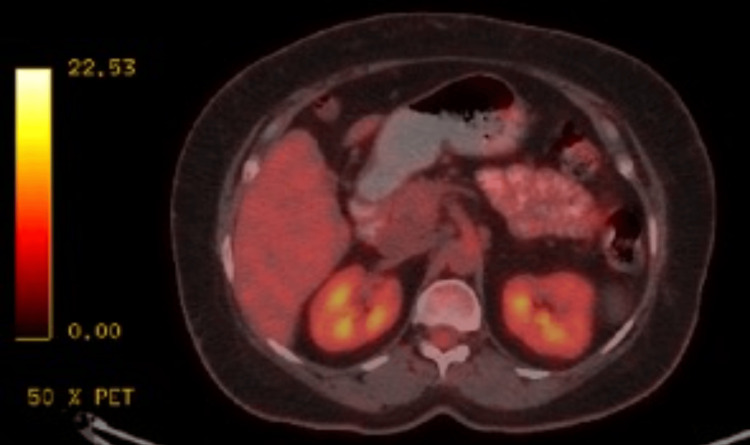
Positron emission tomography/CT showed a complete metabolic response at sites of previous lymphoma involvement.

After her transplant course was complicated by febrile neutropenia that was treated with IV antibiotics, she was engrafted in a timely manner and discharged home. She came for a follow-up and was well, with a gradual improvement in performance status after high-dose therapy. Response evaluation around three months post-ASCT, PET/CT showed a complete metabolic response at sites of previous lymphoma involvement with no PET/CT evidence of disease progression or new sites of involvement, which was a Deauville score 1 (Figure 7). However, metabolically active small left superficial inguinal and left external iliac lymph nodes occurred, which were favored to represent reactive lymph nodes.

The patient was continued on pembrolizumab maintenance for six months afterward. Currently, the patient is healthy and doing well. Furthermore, approximately six months post-ASCT, the patient received her initial dose of the COVID-19 vaccine as per EBMT guidelines and then received two more doses afterward without any complications.

## Discussion

The current understanding is limited as to the outcome of COVID-19 infection in Hodgkin’s lymphoma cases and whether chemotherapeutic agents or the immunosuppressive state can worsen the patient’s outcome [17]. According to a report from the Centers for Disease Control and Prevention (CDC), patients with weakened immune systems, such as those who have had cancer treatment, bone marrow or organ transplants, face the highest risk of experiencing severe COVID-19 [18]. This is supported by other reports that revealed that individuals with cancer are at an elevated risk of experiencing severe illness as a result of COVID-19 compared to the general population [19]. However, managing hematological malignancy patients with COVID-19 infection is highly individualized and requires more case studies to gain a better understanding of how to handle such situations.

The case described herein presents the disease course of a refractory Hodgkin’s lymphoma patient who started a modified chemotherapy course without completely recovering from the COVID-19 infection (shedding of infection). Thus, she was given dexamethasone. However, for patients who are too sick or immunocompromised, there may be an alternative, such as Evusheld, a combination of cilgavimab and tixagevimab. However, it has demonstrated some limited efficacy against omicron and was granted emergency use authorization (EUA) status by the Food and Drug Administration (FDA); consequently, it might find application as “pre-exposure” in some high-risk cancer patients. Nevertheless, it is not authorized for treatment or post-exposure prophylaxis, and patients must fulfill specific criteria outlined by the FDA to qualify for Evusheld [20].

Depending on individual cases, the first-line treatment of classical Hodgkin’s lymphoma is either an ABVD or Bv-AVD chemotherapy regimen [21]. Medical oncology communities struggle to provide suitable care to their cancer patients, and they are obligated to stop or manipulate chemotherapy during the course of the disease. Moreover, oncologists have developed treatment modifications in order to balance the advantage of refining cancer-related outcomes with the risk of contracting the virus. Given that our patient, in this case report, is COVID-19 positive, she was given dexamethasone as well as received the front-line therapy of a CD30-directed antibody-drug conjugate (brentuximab) plus AVD without Bleomycin since it might have a negative effect on the outcome in Hodgkin’s lymphoma patients as it may cause lung toxicity [22,23]. A review of the clinical characteristics and outcomes of malignancy patients in New York City concluded that hematological cancer is concomitant with the augmented severity of COVID-19 [24]. Similarly, this patient suffered from unfavorable symptoms before chemotherapy/biological therapy.

Previous studies reported lymphopenia as the most common laboratory finding associated with COVID-19 cases [25-27]. On the other hand, chemotherapy was also reported to result in severe lymphopenia and neutropenia [28,29]. Moreover, Echelon-1 clinical trials showed that using brentuximab resulted in lymphopenia and neutropenia in most of their patients, and prophylaxis with GCSF is recommended [21]. Likewise, our patient developed lymphopenia and neutropenia following the use of brentuximab. However, luckily, brentuximab did not cause any severe adverse effects on the disease.

The patient presented here has failed the first-line treatment of antibody-drug conjugate therapy as well as the salvage therapy since new legions have developed, as shown in the PET scan. It has been demonstrated that unresponsive adult patients to at least one line of chemotherapy and/or salvage therapy are candidates for pembrolizumab (a PD-1 inhibitor) preceding ASCT [30]. Therefore, in line with the previous study, the patient in this report was started on immune-engage therapy (pembrolizumab), which successfully resolved the previous pelvis and abdominal lymph nodes.

Research has demonstrated that a patient with classical Hodgkin’s lymphoma showed complete remission while infected with SARS-CoV-2. More recently, monoclonal antibodies, such as pembrolizumab targeting PD-1, have been found to increase the overall survival of classical Hodgkin’s lymphoma patients. By binding to the PD-L1 binding site of PD-1 and blocking the access of PD-L1 and PD-L2, they induce apoptosis and prevent the relapse of classical Hodgkin’s lymphoma. Some coronavirus infections induce apoptosis in host cells, which may be required for viral replication and propagation in their respective hosts. However, the precise molecular mechanism of this rare event is yet to be understood [31].

Generally, therapeutic choices are restricted for patients who relapse after salvage and targeted chemotherapy [32]. Previous randomized trials demonstrated that the ASCT approach is effective and safe for relapsed and resistant Hodgkin's disease in a dose-dependent manner [33,34]. In the current case, oncologists have decided that the patient is a candidate for high-dose therapy and then ASCT. Fortunately, she survived the transplantation. Since there is no standard treatment (single or combination therapy regimens) following ASCT [30], our patient was given pembrolizumab for maintenance as she responded to this immunotherapy earlier during the course of the disease. Some individuals may benefit from maintenance therapy with pembrolizumab, a PD-1 inhibitor, following ASCT for refractory/relapsed classic Hodgkin’s lymphoma. In classic Hodgkin’s lymphoma, Hodgkin Reed-Sternberg (HRS) cells overexpress the PD-1 ligands PD-L1 and PD-L2, which allows them to avoid immune surveillance. With PD-1 inhibitors, small prospective studies have revealed high response rates. However, more research is needed to define the treatment's efficacy [35].

## Conclusions

In conclusion, the relapsed/refractory Hodgkin’s lymphoma case presented here shows a good outcome using immune-engaged therapy followed by HDT/ASCT. Her oncologist had to change the front-line chemotherapy regimens due to the COVID-19 infection to avoid lung toxicity. Consequently, she showed minimal therapy-related complications with the recommended treatments. Unfortunately, there is inadequate data on COVID-19 among lymphoma patients, with few described cases. Thus, further studies are necessary to assess the therapeutic effect of chemotherapy doses and combinations on a large scale in refractory/relapsed Hodgkin’s lymphoma patients.
